# Colquhounia root tablet improves diabetic kidney disease by regulating epithelial-mesenchymal transition via the PTEN/PI3K/AKT pathway

**DOI:** 10.3389/fphar.2024.1418588

**Published:** 2024-07-26

**Authors:** Donghong Ma, Jiao Zhang, Lu Du, Jingjing Shi, Zhaoyan Liu, Jilin Qin, Xiaoxiao Chen, Minghao Guo

**Affiliations:** ^1^ Department of Nephrology, The First Affiliated Hospital of Xinxiang Medical University, Weihui, Henan Province, China; ^2^ Xinxiang Key Laboratory of Precise Therapy for Diabetic Kidney Disease, The First Affiliated Hospital of Xinxiang Medical University, Weihui, Henan Province, China

**Keywords:** Colquhounia root tablet, diabetic kidney disease, epithelial-mesenchymal transition, pten, PI3K/Akt pathway

## Abstract

**Background:**

Diabetic kidney disease (DKD) is a severe microvascular complication of diabetes mellitus that can lead to end-stage renal disease. Colquhounia root tablet (CRT) has shown therapeutic potential in treating DKD, but its efficacy and underlying mechanisms remain to be elucidated.

**Methods:**

A randomized controlled clinical trial was conducted on 61 DKD patients. The treatment group received CRT in addition to standard therapy, while the control group received standard therapy alone. Treatment efficacy and adverse events were evaluated after 3 months. Additionally, *in vitro* experiments using human renal tubular epithelial cells (HK-2) were performed to investigate the effect of CRT on high glucose (HG)-induced epithelial-mesenchymal transition (EMT) and the involvement of the PTEN/PI3K/AKT signaling pathway.

**Results:**

CRT treatment significantly improved proteinuria and increased the effective treatment rate in DKD patients compared to the control group, with no significant difference in adverse events. Moreover, CRT reversed HG-induced EMT in HK-2 cells, as evidenced by the downregulation of α-SMA and upregulation of E-cadherin at both mRNA and protein levels. Mechanistically, CRT increased PTEN expression and inhibited the PI3K/AKT pathway, similar to the effects of the PI3K inhibitor LY29400. The combination of CRT and LY29400 further enhanced PTEN mRNA expression under HG conditions.

**Conclusion:**

CRT effectively improves proteinuria in DKD patients and ameliorates HG-induced EMT in HK-2 cells. The underlying mechanism may involve the upregulation of PTEN and subsequent inhibition of the PI3K/AKT signaling pathway. These findings provide new insights into the therapeutic potential of CRT for DKD treatment.

## 1 Introduction

Diabetic kidney disease (DKD), a microvascular complication caused by diabetes mellitus (DM), can progress to end-stage renal disease (ESRD), accounting for approximately one-third of ESRD cases (Reutens and Atkins, 2011). This microvascular complication affects about 30% of patients with type 1 diabetes mellitus (DM1) and approximately 40% of those with type 2 diabetes mellitus (DM2) (Saran et al., 2018; Hoogeveen, 2022). As the prevalence of DM increases, the incidence of DKD continues to grow (de Boer et al., 2011). Among all chronic diseases, DKD is one of the leading causes of increased mortality in recent years (Jha et al., 2013). Moreover, treatment costs escalate as DKD progresses, placing a heavy economic burden on patients (Golestaneh et al., 2017).

Numerous studies have substantiated the therapeutic value of *Tripterygium wilfordii* (TW) preparations for DKD. A meta-analysis encompassing 19 systematic reviews and meta-analyses revealed that the most common TW preparation, *T. wilfordii* polyglycoside (TWP), effectively improves proteinuria and serum albumin levels in DKD patients (Wang K. et al., 2022). The potential pharmacological mechanisms of TW have also been thoroughly explored and validated (Wang et al., 2020; Lu et al., 2023). Colquhounia Root Tablet (CRT) represents a new type of TW, derived from the root of Tripterygium hypoglaucum (H. L’ev.) Hutch. Its active ingredients include alkaloids such as triptolide, terpenes such as tripterine, vinegar components like aristolochic acid, and phenolic acids such as tripterinin (Zhou et al., 2018). CRT possesses high clinical value, with studies confirming its efficacy and low toxicity in treating rheumatoid arthritis, DKD, and membranous nephropathy (Wang Y. et al., 2022). CRT exhibits favorable anti-inflammatory, analgesic, and immunosuppressive pharmacological properties. As immune responses and inflammatory reactions are involved in the pathogenesis of DKD, CRT can be considered a candidate drug for DKD treatment (Gnudi et al., 2016; Lv et al., 2018; Feng et al., 2022). Recent studies using DKD rat models and human kidney-2 cells (HK-2) have explored the pharmacological properties and potential mechanisms of CRT in treating DKD *in vitro*, fully predicting its clinical therapeutic potential (Ma et al., 2022).

Epithelial-mesenchymal transition (EMT) refers to the process by which epithelial cells gradually transform into mesenchymal cells (Kalluri and Weinberg, 2009). Research has found that tubulointerstitial fibrosis plays a crucial role in the pathogenesis of DKD, with EMT in renal tubular epithelial cells being the most common cause of renal fibrosis and impaired renal function in DKD patients (Loeffler et al., 2018). Studies have substantiated the pivotal roles of α-smooth muscle actin (α-SMA) and E-cadherin in the EMT process, establishing them as reliable biomarkers for EMT progression (Ding et al., 2014; Sommariva and Gagliano, 2020). The upregulation of α-SMA expression and concomitant downregulation of E-cadherin expression are widely regarded as indicative of the advancement of EMT. The PI3K/Akt signaling pathway is closely related to extracellular matrix (ECM) accumulation and tubulointerstitial fibrosis in DKD. Abnormal activation of AKT is involved in the occurrence of high glucose (HG)-induced EMT in HK-2 (Xie et al., 2015; Xu et al., 2015). The phosphatase and tensin homolog (PTEN) gene, located on chromosome 10, was identified by Li in 1997 through a detailed study of the 10q23 region. The PTEN protein, consisting of 403 amino acids, exhibits dual phosphatase activity for both proteins and lipids, and plays a critical role in various biological functions (Li et al., 1997). In signal transduction processes, PTEN negatively regulates the PI3K/Akt signaling pathway (Ramaswamy et al., 1999).

This study is the first to employ a comprehensive randomized controlled clinical trial to verify that CRT can improve proteinuria in DKD patients and explore its efficacy. Furthermore, based on previous research findings that CRT improves DKD through the PI3K/Akt pathway, this study aims to further investigate and elucidate the impact of CRT on EMT and the PTEN/PI3K/AKT pathway, providing new theoretical support for CRT treatment of DKD.

## 2 Methods

### 2.1 Study population

A total of 61 patients with DKD admitted to the Department of Nephrology, the First Affiliated Hospital of Xinxiang Medical University, from May 2021 to October 2022 were selected as the study subjects. The inclusion criteria were as follows: 1) a clear history of diabetes; 2) clinical or renal pathological diagnosis of DKD; the diagnostic criteria for DKD were based on the 2021 Chinese guidelines (Wp, 2018); 3) age between 18 and 75 years; and 4) a 24-h urine protein quantification >300 mg and/or an estimated glomerular filtration rate (eGFR) < 60 mL/min/1.73 m^2^. Patients were excluded from the study if they met any of the following criteria: 1) presence of infection, tumors, or severe cardiopulmonary or liver dysfunction; 2) concurrent acute complications of diabetes; 3) psychiatric disorders; or 4) pregnancy or lactation in female patients. This study was approved by the hospital’s medical ethics committee, and all subjects enrolled in the study signed informed consent forms (Ethics approval number: 2020106).

### 2.2 Study design and treatment protocol

The patients were divided into two groups based on whether they received additional treatment with CRT (manufactured by Chongqing Pharmaceutical Research Institute Co., Ltd.; specification: 0.18 g; approval number: Z20027411): the control group (31 cases) and the CRT group (30 cases). Both groups received standard DKD treatment, including dietary habit changes (diabetic diet and high-quality low-protein diet), lifestyle interventions (appropriate physical exercise, smoking cessation, and alcohol abstinence), and pharmacological therapies. Blood glucose was controlled using metformin, sulfonylureas, and insulin; blood pressure was managed with angiotension converting enzyme inhibitors (ACEIs) or angiotensin II receptor blockers (ARBs); and lipid levels were lowered with statins such as atorvastatin and simvastatin.

At the beginning of the treatment, patients in the CRT group were given four tablets per dose, three times per day. After 1 month of medication, if no obvious adverse reactions occurred and the 24-h urine protein quantification reduction was <30%, the dosage was increased to five tablets per dose,three3 times per day. If adverse reactions such as gastrointestinal reactions or liver function damage occurred, the dosage was reduced to three tablets per dose, three times per day. Each tablet contains 4 μg of triptolide and 0.2 mg of epigallocatechin, which are the main bioactive compounds in CRT.

### 2.3 Data collection and measurement

The following data were collected: sex, age, body mass index (BMI), duration of diabetes, presence of DKD, blood pressure (BP), 24-h urine protein quantification, hemoglobin (Hb), blood urea nitrogen (BUN), creatinine (Cr), eGFR, uric acid (UA), glucose (Glu), glycosylated hemoglobin A1c (HbA1c), cholesterol (CHO), triglyceride (TG), high-density lipoprotein cholesterol (HDL-CHO), low-density lipoprotein cholesterol (LDL-CHO), albumin (Alb), alanine transaminase (ALT), aspartate transaminase (AST), and the use of angiotensin-converting enzyme inhibitors (ACEI) or angiotensin II receptor antagonists (ARB).

### 2.4 Follow-up and endpoint definition

Patients in both groups received continuous medication for 3 months and were followed up at the hospital once a month. In the event of any adverse reactions or critical illnesses during the medication period, appropriate treatment was promptly provided or the medication was discontinued.

The primary endpoint of the study was treatment effectiveness, defined as an improvement in clinical symptoms and a reduction in 24-h urine protein quantification by 30% or greater. The treatment effectiveness rates of the two groups were compared at the end of the 3-month treatment period. Adverse events occurring during the medication period were monitored and recorded to assess the safety profile of the interventions.

### 2.5 *In vitro* experimental validation

HK-2 cells (#CL-0109, Procell Life Science & Technology Co., Ltd., Wuhan, China) were uniformly seeded in T25 flasks containing 5 mL of complete culture medium (10% fetal bovine serum, 90% MEM, 100 U/mL penicillin-streptomycin) and cultured at 37°C with 5% CO_2_. The culture medium was changed every 2 days. When cells reached 90% confluency, they were passaged at a 1:2 ratio. Cells from the third passage with a viability above 95% were used for experiments.

The cells were divided into the following groups: CON group (glucose concentration 5.5 mmol/L in the culture medium), MA group (glucose concentration 5.5 mmol/L, mannitol concentration 24.5 mmol/L in the culture medium), HG group (glucose concentration 30 mmol/L in the culture medium), HG + CRT group (glucose concentration 30 mmol/L, CRT(#221002, Pharmaceutical Research Institute Co., Ltd., Chongqing, China) concentration 50 ug/mL in the culture medium), HG + PI3K inhibitor group (glucose concentration 30 mmol/L, LY29400 (#10494-1-AP, Sanying Biotechnology Co., Ltd., Wuhan, China) concentration 20 umol/L in the culture medium), and HG + CRT + PI3K inhibitor group (glucose concentration 30 mmol/L, CRT concentration 50 ug/mL, LY29400 concentration 20 umol/L in the culture medium). The cells were cultured for 48 h before subsequent experiments.

### 2.6 RT-PCR analysis

The mRNA expression levels of α-SMA, E-cadherin, and PTEN in cells from each group were determined using RT-PCR (total RNA extraction reagent (Trizol): #341446AX, Invitrogen, United States; reverse transcription kit: #E096-01A, Novoprotein Scientific Co., Ltd., Shanghai, China; PCR kit: #E047-01B, Novoprotein Scientific Co., Ltd., Shanghai, China). Total RNA was extracted from the CON, MA, HG, HG + CRT, HG + LY29400, and HG + CRT + LY29400 groups using the Trizol method, and cDNA was obtained using a reverse transcription kit. The primer sequences for α-SMA, E-cadherin, PTEN, and GAPDH are provided in the supplementary materials. The reaction system (20 μL) consisted of 10 μL 2×NovoStart^®^ SYBR qPCR SuperMix Plus, 2 μL each of upstream and downstream primers, 2 μL template cDNA, 0.4 μL ROX Reference Dye II (ROX II), and RNase-Free Water to a total volume of 20 μL. The amplification conditions were as follows: pre-denaturation at 95°C for 1 min, denaturation at 95°C for 20 s, and annealing at 60°C for 1 min, for a total of 45 cycles. Three independent sample experiments were performed for each group.

### 2.7 Immunofluorescence staining

The expression of α-SMA and E-cadherin proteins in cells from each group was assessed using immunofluorescence staining (enhanced chemiluminescence (ECL) chemiluminescence kit: #BMU102-CN, Abbkine Scientific Co., Ltd., Units). Cell-climbing slices from each group were washed with phosphate-buffered saline (PBS) and fixed with a 4% paraformaldehyde solution for 20 min. After washing, the cells were permeabilized with 0.2% Triton X-100 for 10 min. Following another wash, the cells were blocked with a bovine serum albumin (BSA) solution for 30 min. The slices were then incubated with diluted primary antibodies against α-SMA and E-cadherin in a humid box at 4°C overnight. After washing with PBS, the corresponding fluorescent secondary antibodies were added, and the slices were incubated at room temperature for 1 h in the dark. The slices were then washed with PBS and stained with 4′,6-diamidino-2-phenylindole (DAPI) for 10 min, avoiding light exposure. Finally, the slices were mounted with an anti-quenching agent and photographed under a fluorescence microscope.

### 2.8 Western blot analysis

The expression levels of PTEN, PI3K, AKT, p-AKT, E-cadherin, and α-SMA proteins in cells from each group were determined using Western blotting (α-SMA rabbit monoclonal antibody: #19245, PTEN rabbit monoclonal antibody: #9188, PI3K rabbit monoclonal antibody: #4249, AKT rabbit monoclonal antibody: #4691, p-AKT rabbit monoclonal antibody: #4060, Cell Signaling Technology, Inc., United States; E-cadherin rabbit monoclonal recombinant antibody: #19245, HuaAn Biotechnology Co., Ltd., Hangzhou, China; GAPDH rabbit polyclonal antibody: #10494-1-AP, Sanying Biotechnology Co., Ltd., Wuhan, China). Cells were lysed on ice, and protein concentrations of the samples were measured using the BCA method (BCA kit: KTD3001, Abbkine Scientific Co., Ltd., United States). The samples were then fully denatured in a 100°C water bath for 10 min. The denatured protein samples were separated by 10% sodium dodecyl sulfate-polyacrylamide gel electrophoresis (SDS-PAGE) and transferred to polyvinylidene fluoride (PVDF) membranes. The membranes were blocked with 5% skim milk for 90 min and incubated with primary antibodies overnight at 4°C. Subsequently, the membranes were incubated with secondary antibodies for 1 h at room temperature. After washing with 1× Tris-buffered saline with Tween-20 (TBST), the membranes were exposed using a scanner, and the gray values were quantified using ImageJ software.

### 2.9 Statistical analyses

Normally distributed continuous variables were expressed as the mean ± standard deviation (X ± S). The data were tested for normality and homogeneity of variance. If the conditions were met, one-way analysis of variance (ANOVA) was used for comparisons among multiple groups. If the variances were unequal, non-parametric tests were employed. For non-normally distributed continuous variables, data were presented as median and interquartile range M (Q1, Q3), and comparisons between groups were performed using non-parametric tests. Categorical variables were expressed as frequencies or percentages, and comparisons between groups were conducted using the chi-square test. Independent sample t-tests were used for comparisons between groups, while paired *t*-tests were used for within-group comparisons before and after treatment. A *P*-value < 0.05 was considered statistically significant. The data were analyzed using SPSS 23.0 and GraphPad Prism 8.

## 3 Results

### 3.1 Baseline characteristics of study participants

The control group consisted of 19 males and 12 females, with a mean age of 53.68 ± 12.86 years, median diabetes duration of 136 (38, 208) months, systolic blood pressure of 148.16 ± 20.81 mmHg, diastolic blood pressure of 86.39 ± 13.15 mmHg, and median eGFR of 41.74 (25.70, 68.80) mL/min/1.73 m^2^. The CRT group included 17 males and 13 females, with a mean age of 52.47 ± 10.47 years, median diabetes duration of 126 (54, 190) months, systolic blood pressure of 154.73 ± 23.11 mmHg, diastolic blood pressure of 90.77 ± 13.78 mmHg, and median eGFR of 50.66 (27.30, 77.84) mL/min/1.73 m^2^. There were no statistically significant differences in baseline characteristics between the two groups (*P* > 0.05) (Table 1).

**TABLE 1:** Baseline characteristics of study participants.

Parameters	Control group (n = 31)	CRT group (n = 30)	*P*-value
Male, n (%)	19 (61.3)	17 (56.7)	0.714
Age, X ± S, years	53.68 ± 12.86	52.47 ± 10.47	0.689
DR, n (%)	26 (42.6)	23 (37.7)	0.479
Duration of DM,M (Q1, Q3),months	136 (38, 208)	126 (54, 190)	0.583
BMI,X ± S, kg/m2	25.94 ± 3.38	26.72 ± 3.26	0.361
SBP,X ± S, mmHg	148.16 ± 20.81	154.73 ± 23.11	0.247
DBP,X ± S, mmHg	86.39 ± 13.15	90.77 ± 13.78	0.209
eGFR, M (Q1, Q3), mL/min/1.73m2	41.74 (25.70, 68.80)	50.66 (27.30, 77.84)	0.471
FBG, M (Q1, Q3), mmol/L	6.49 (5.94, 7.92)	7.68 (5.84, 9.79)	0.217
HbA1c, M (Q1, Q3), %	6.9 (6.07, 8.16)	7.56 (5.84, 9.79)	0.506
Alb, X ± S, g/L	36.40 ± 5.96	36.62 ± 5.88	0.884
AST, M (Q1, Q3), U/L	16 (11, 21)	16 (13, 21.25)	0.633
ALT, M (Q1, Q3), U/L	16 (9, 22)	17 (11.5, 23.5)	0.448
Hb, M (Q1, Q3), g/L	108 (101, 132)	114.5 (98.5, 126.25)	0.751
SCr, M (Q1, Q3), μmol/L	121.8 (88.8, 216.3)	113.7 (71.63, 187.15)	0.609
BUN, M (Q1, Q3), mmol/L	9.72 (6.62, 13.67)	8.89 (6.82, 12.16)	0.554
UA, M (Q1, Q3), μmol/L	365 (309, 445)	328 (284.5, 380.5)	0.093
TC, M (Q1, Q3), mmol/L	4.7 (3.42, 5.98)	4.51 (3.83, 5.55)	0.988
TG, M (Q1, Q3), mmol/L	1.71 (1.2, 1.93)	2.05 (1.00, 2.87)	0.634
HDL-C, M (Q1, Q3), mmol/L	1.12 (0.85, 1.43)	1.15 (0.96, 1.40)	0.790
LDL-C, M (Q1, Q3), mmol/L	2.75 (1.94, 3.64)	2.44 (2.07, 3.03)	0.493
24 h urine protein, M (Q1, Q3), g	3.98 (1.95, 4.77)	4.42 (2.13, 7.75)	0.267
ACEI/ARB, n (%)	21 (67.74)	21 (70.00)	0.849

Abbreviation: DR, diabetic retinopathy; DM, diabetes mellitus; BMI, body mass index; DKD, diabetic kidney disease; SBP, systolic blood pressure; DBP, diastolic blood pressure; eGFR, estimated glomerular filtration rate; FBG, fasting blood glucose; HbA1c, glycosylated hemoglobin A1c; Alb, albumin; ALT, alanine transaminase; AST, aspartate transaminase; Hb, hemoglobin; BUN, blood urea nitrogen; SCr, serum creatinine; UA, uric acid; TC, total cholesterol; TG, triglyceride; HDL-C, high-density lipoprotein cholesterol; LDL-C, low-density lipoprotein cholesterol; ACEI, angiotensin-converting enzyme inhibitors; ARB, angiotensin II, receptor antagonists.

### 3.2 24-h urine protein decline rates and the treatment effectiveness rates of two groups

24-h urine protein decline rates and the treatment effectiveness rates of two groups are shown in Table 2. 24-h urine protein decline rates are higher in the CRT group than in the control group after treatment for 1, 2, and 3 months, respectively. However, the difference was not significant (*p* > 0.05). The CRT group showed a treatment effectiveness rate of 93.33%, which is significantly higher than that of the control group (*p* < 0.05). In the control group, the adverse event rate was 3.23%, with one patient presenting a liver injury. While in the CRT group, the adverse events rate was 10%, with one patient presenting a liver injury and two patients presenting gastrointestinal adverse reactions. The difference in adverse event rates between the two groups was not significant.

**TABLE 2:** 24 h urine protein decline rates and the treatment effectiveness rate two groups.

Parameters	Control group (n = 31)	CRT group (n = 30)	*P*-value
Baseline 24 h urine protein M (Q1, Q3), g	3.98 (1.95, 4.77)	4.42 (2.13, 7.75)	0.428
1M 24 h urine protein M (Q1, Q3), g	3.44 (2.90, 6.63)	3.67 (1.68, 5.46)	0.908
1M 24 h urine protein decline rate, M, %	16%	22%	0.614
2M 24 h urine protein, M (Q1, Q3), g	3.34 (1.73, 5.18)	3.29 (1.89, 4.63)	0.708
2M 24 h urine protein decline rate, M, %	26%	34%	0.428
3M 24 h urine protein, M (Q1, Q3), g	3.22 (2.40, 5.91)	2.68 (2.03, 3.45)	0.535
3M 24 h urine protein decline rate, M, %	41%	51%	0.507
Treatment effectiveness rates, n (%)	22 (70.97%)	28 (93.33%)	0.023
Adverse events rates, n (%)	1 (3.23%)	3 (10%)	0.581
Liver injury, n (%)	1 (3.23%)	1 (3.33%)	
Gastrointestinal adverse reactions, n (%)	0 (0%)	2 (6.67%)	

### 3.3 Comparison of biochemical parameters between two groups

Comparisons of biochemical parameters between two groups are shown in Table 3. The CRT group showed a higher level of HbA1c and FBG and a lower level of SCr, BUN, and TC after 3 months of treatment than the control group, but the difference was not significant (*P* > 0.05). Baseline Alb was slightly higher in the CRT group than in the control group. After 3 months of treatment, Alb showed a significantly higher level in the CRT group than the control group (*P* = 0.025).

**TABLE 3:** Comparison of biochemical parameters between two groups.

Parameters	Control group (n = 31)	CRT group (n = 30)	*P*-value
SCr,M (Q1, Q3), μmol/L			
Baseline	121.8 (88.8, 216.3)	113.7 (71.63, 187.15)	0.609
After 3M treatment	124.1 (86.3, 196)	109.6 (89.42, 200)	0.535
BUN,M (Q1, Q3), mmol/L			
Baseline	9.72 (6.62, 13.67)	8.89 (6.82, 12.16)	0.554
After 3M treatment	9.51 (7.22, 19.94)	8.77 (7.30, 12.33)	0.348
Alb,X ± S, g/L			
Baseline	36.40 ± 5.96	36.62 ± 5.88	0.884
After 3M treatment	36.55 ± 6.57	40.10 ± 5.39	0.025
FBG,M (Q1, Q3), mmol/L			
Baseline	6.49 (5.94, 7.92)	7.68 (5.84, 9.79)	0.217
After 3M treatment	6.07 (5.62, 7.46)	7.18 (5.78, 8.37)	0.093
HbA1c,M (Q1, Q3), %			
Baseline	6.9 (6.07, 8.16)	7.56 (5.84, 9.79)	0.506
After 3M treatment	6.77 (5.80, 7.83)	6.81 (5.81, 7.25)	0.885
TC,M (Q1, Q3), mmol/L			
Baseline	4.7 (3.42, 5.98)	4.51 (3.83, 5.55)	0.988
After 3M treatment	4.47 (3.42, 5.68)	4.47 (3.42, 5.68)	0.801

Abbreviation: FBG, fasting blood glucose; HbA1c, glycosylated hemoglobin A1c; Alb, albumin; BUN, blood urea nitrogen; SCr, serum creatinine; TC, total cholesterol.

### 3.4 CRT reverses HG-induced changes in α-SMA and E-cadherin expression

**FIGURE 1:**
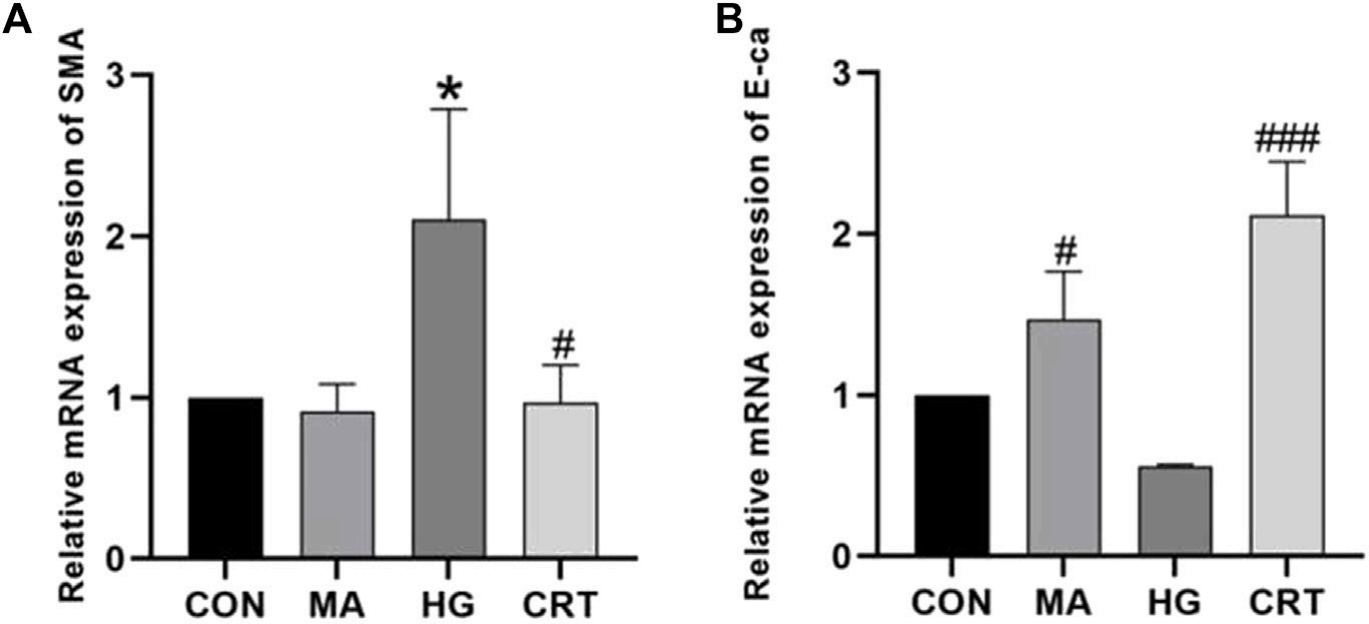
**(A)** Relative mRNA expression levels of α-SMA between the groups; **(B)** Relative mRNA expression levels of E-cadherin between the groups. Data are shown as the mean ± SD. #*p* < 0.05, ##*p* < 0.01, ###*p* < 0.001, comparison with the HG group; *p < 0.05, **p < 0.01, ***p < 0.001, comparison with the CON group.

Compared to the control group, HK-2 cells stimulated with HG for 48 h exhibited increased mRNA expression levels of α-SMA and decreased mRNA expression levels of E-cadherin (*P* < 0.05). The MA group showed no significant changes. However, the addition of CRT reversed these alterations (*P* < 0.05) (Figure 1).

### 3.5 CRT and LY29400 increase PTEN expression in HG-induced HK-2 cells

**FIGURE 2:**
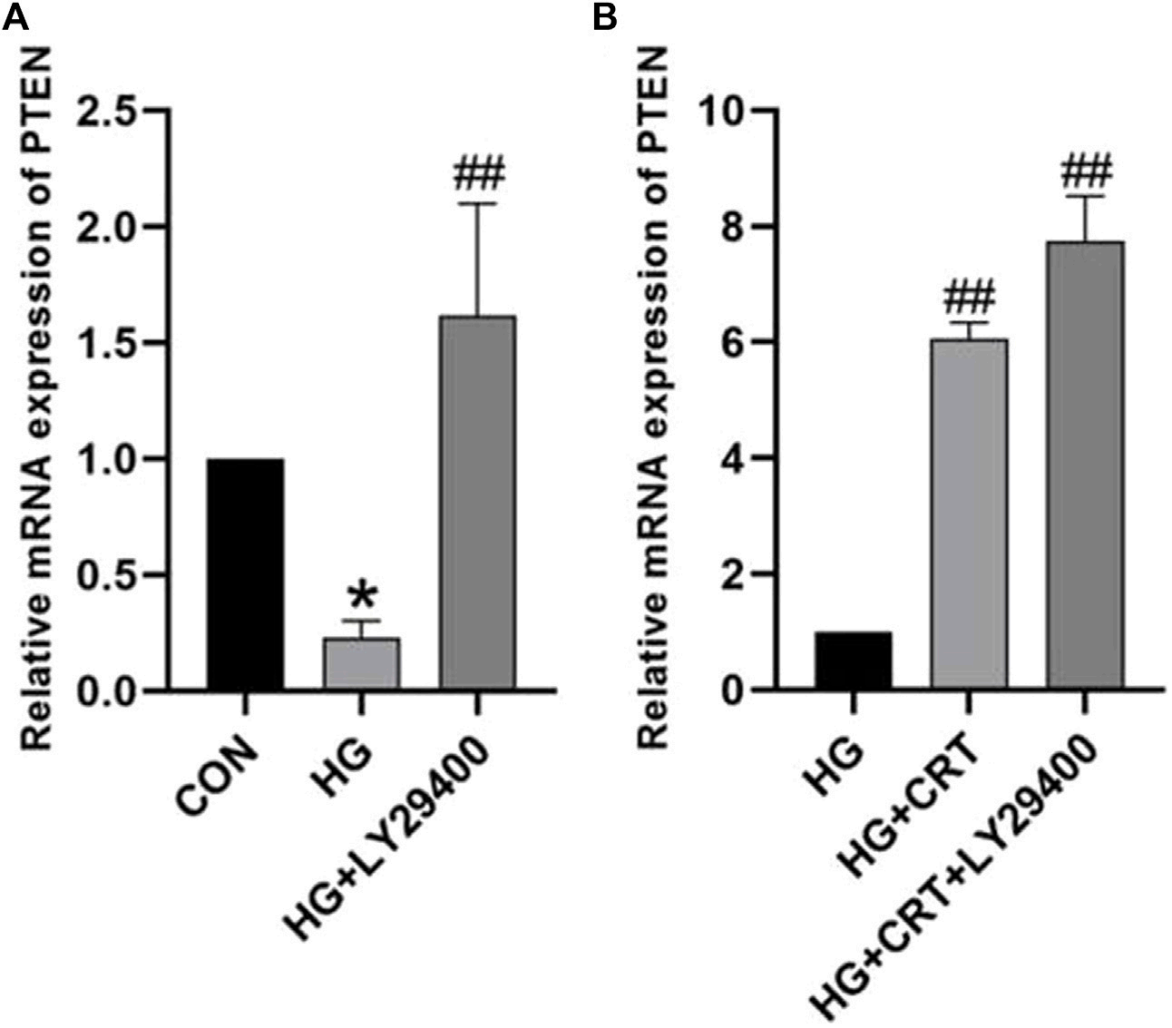
**(A)** Relative mRNA expression levels of PTEN between the CON group, HG group, and HG + LY24900 group; **(B)** Relative protein expression levels of PTEN between the HG group, HG + CRT group, and HG + CRT + LY24900 group. Data are shown as the mean ± SD. #*p* < 0.05, ##*p* < 0.01, ###*p* < 0.001, comparison with the HG group; *p < 0.05, **p < 0.01, ***p < 0.001, comparison with the CON group.

Compared to the control group, HK-2 cells stimulated with HG for 48 h showed decreased mRNA expression levels of PTEN. Intervention with LY29400 resulted in increased PTEN mRNA expression (*P* < 0.05). Similarly, the HG + CRT group exhibited increased mRNA expression levels of PTEN mRNA compared to the HG group, and the use of LY29400 further elevated PTEN mRNA expression levels (*P* < 0.05) (Figure 2).

### 3.6 Immunofluorescence staining results of E-cadherin and α-SMA in HK-2 cells

**FIGURE 3:**
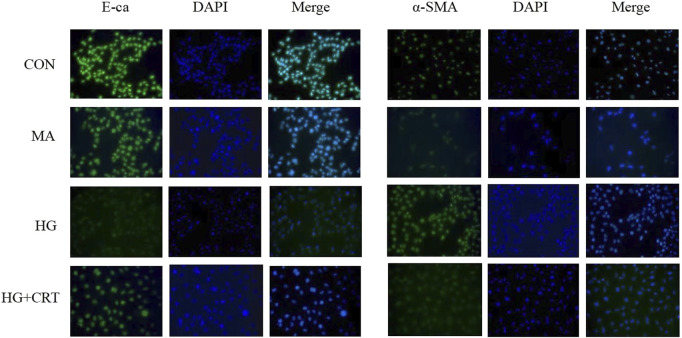
Effect of HG and CRT on the expression levels of EMT marker proteins in HK-2 (100X).

**FIGURE 4:**
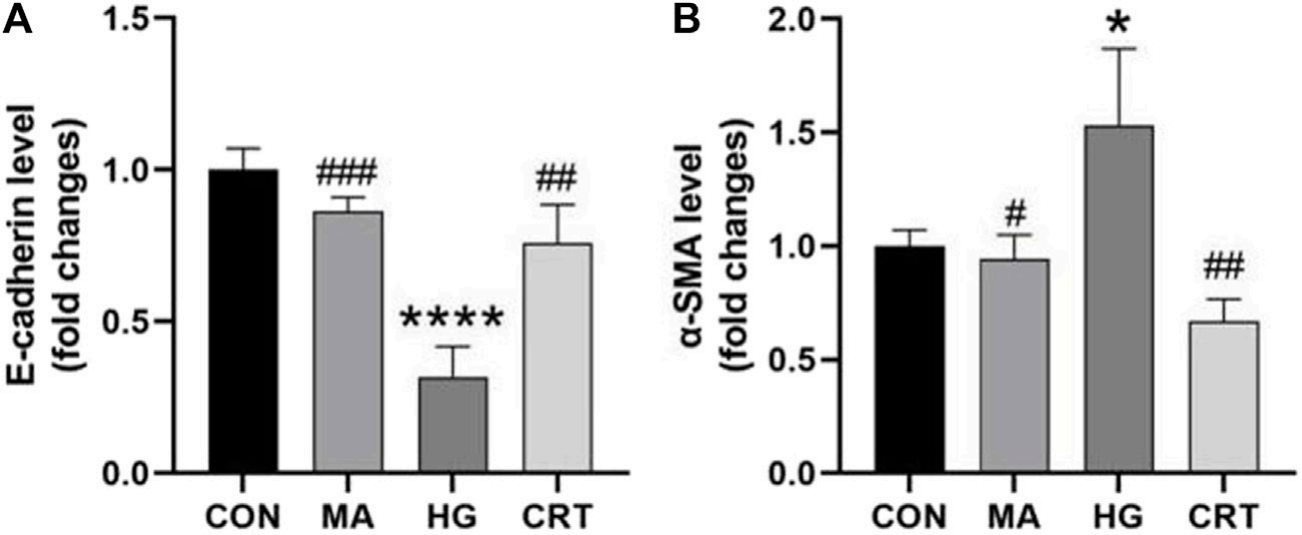
**(A)** Relative protein expression levels of E-cadherin between the groups; **(B)** Relative protein expression levels of α-SMA between the groups. Data are shown as the mean ± SD. #*p* < 0.05, ##*p* < 0.01, ###*p* < 0.001, comparison with the HG group; *p < 0.05, **p < 0.01, ***p < 0.001, comparison with the CON group.

As shown in Figures 3, 4, compared to the control group, HK-2 cells stimulated with HG for 48 h showed increased α-SMA expression and decreased E-cadherin expression (*P* < 0.05). The MA group exhibited no significant changes. However, the addition of CRT (50 ug/mL) significantly reduced α-SMA expression and relatively increased E-cadherin expression (*P* < 0.05).

### 3.7 Comparison of α-SMA and E-cadherin protein expression in HK-2 cells

**FIGURE 5:**
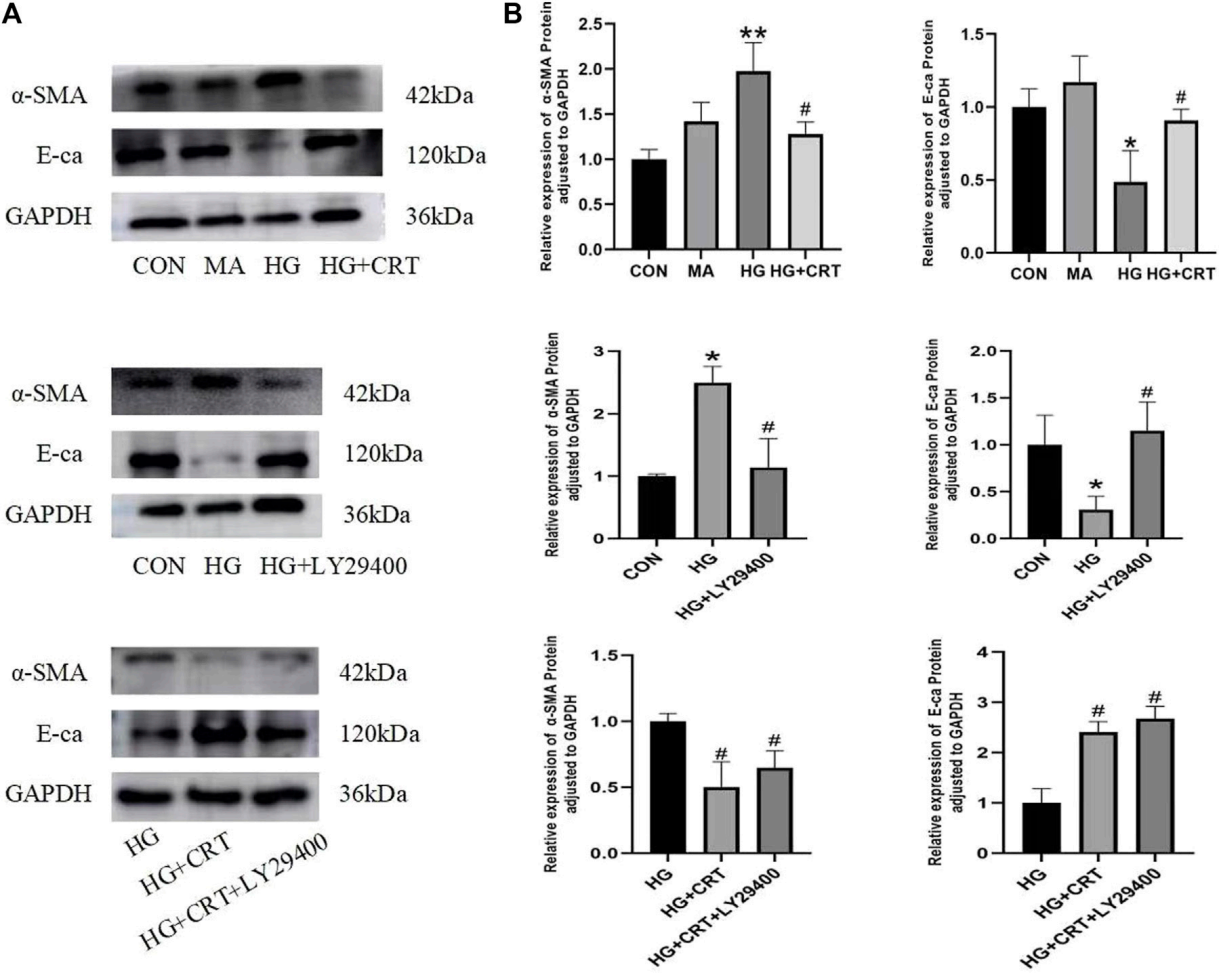
**(A)** Representative western blots of α-SMA, E-cadherin, GAPDH; **(B)** Relative protein expression levels of α-SMA, E-cadherin between the groups. Data are shown as the mean ± SD. #*p* < 0.05, ##*p* < 0.01, ###*p* < 0.001, comparison with the HG group; *p < 0.05, **p < 0.01, ***p < 0.001, comparison with the CON group.

Compared to the control group, the MA group showed no significant differences in E-cadherin and α-SMA protein expression in HK-2 cells. In the HG group, E-cadherin protein expression was significantly decreased (*P* < 0.05), while α-SMA protein expression was significantly increased (*P* < 0.05). However, the HG + LY29400 and HG + CRT groups reversed these changes in protein expression (*P* < 0.05) (Figure 5).

### 3.8 Comparison of PI3K, p-AKT, AKT, and PTEN protein expression in HK-2 cells

**FIGURE 6:**
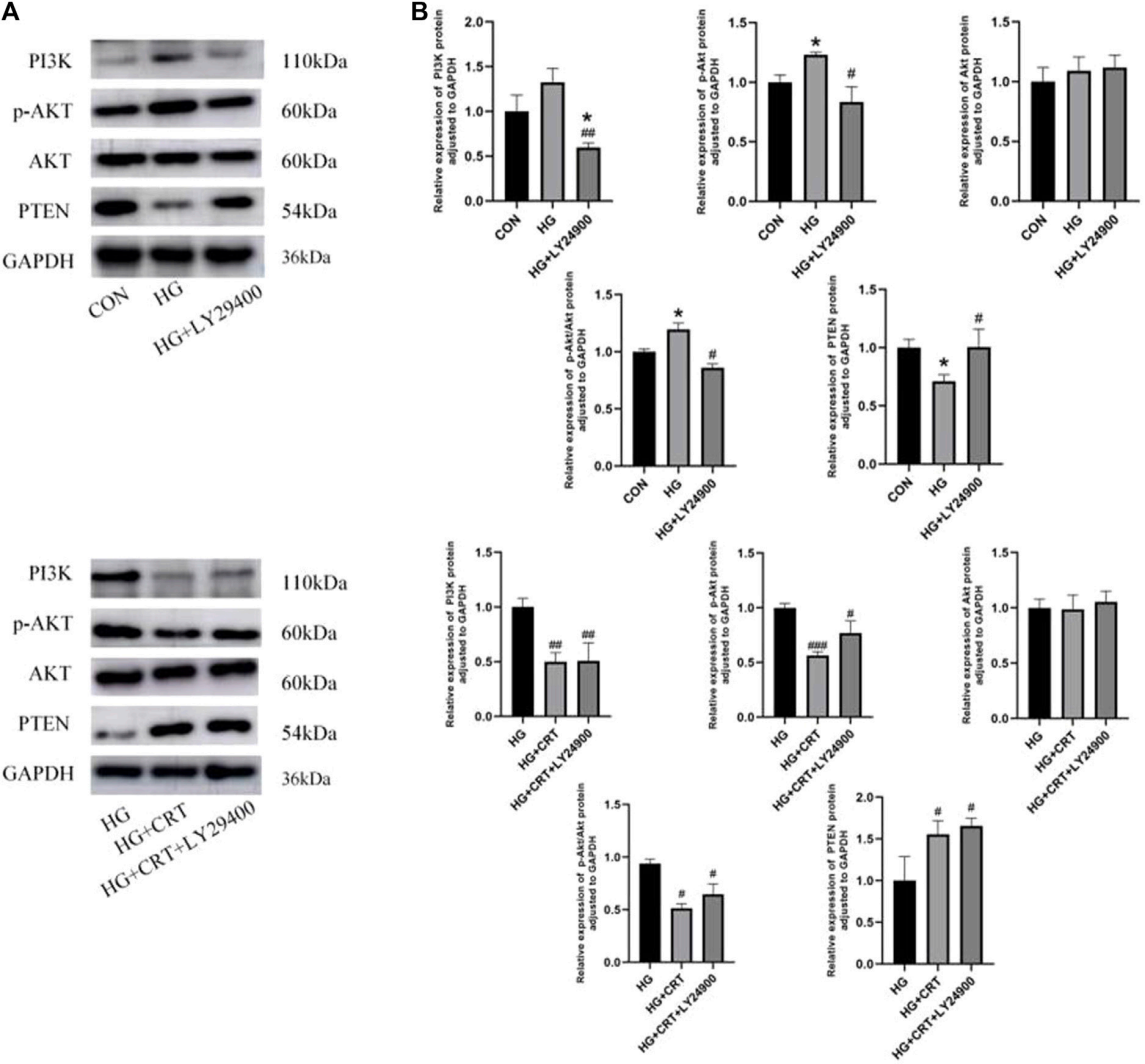
**(A)** Representative western blots of PI3K, p-AKT, AKT, PTEN, GAPDH; **(B)** Relative protein expression levels of PI3K, p-AKT, AKT, p-AKT/AKT, PTEN between the groups. Data are shown as the mean ± SD. #*p* < 0.05, ##*p* < 0.01, ###*p* < 0.001, comparison with the HG group; *p < 0.05, **p < 0.01, ***p < 0.001, comparison with the CON group.

Compared to the control group, the HG group showed no significant changes in PI3K and Akt protein expression. However, p-Akt protein expression was increased (*P* < 0.05), and the p-Akt/Akt protein expression ratio also exhibited an increasing trend (*P* < 0.05). In contrast, PTEN protein expression was decreased (*P* < 0.05). When compared to the HG group, the HG + LYZ9400 group showed no significant changes in Akt protein expression, but PI3K and p-Akt protein expression were decreased (*P* < 0.05). The p-Akt/Akt expression ratio also showed a decreasing trend (*P* < 0.05), while PTEN expression was significantly increased (*P* < 0.05) (Figure 6).

Compared to the HG group, the HG + CRT and HG + CRT + LY29400 groups exhibited no significant changes in Akt protein expression. However, PI3K and p-Akt protein expression were reduced (*P* < 0.05), and the p-Akt/Akt expression ratio was also decreased (*P* < 0.05). PTEN expression was increased (*P* < 0.05) in these groups. When comparing the HG + CRT + LY29400 group to the HG + CRT group, there were no significant differences in E-cadherin, α-SMA, PTEN, PI3K, Akt, and p-Akt/Akt protein expression, but p-Akt protein expression was elevated (*P* < 0.05) (Figure 6).

## 4 Discussion

This study employed a randomized controlled clinical trial and in-depth basic research to validate the therapeutic effects of CRT on patients with DKD and its ameliorative effects on proteinuria and to further explore the potential mechanisms. After 3 months of treatment, we discovered that the efficacy of CRT in treating DKD was significantly higher than that of the control group, with no significant difference in the incidence of adverse events. Moreover, DKD patients treated with CRT exhibited markedly elevated levels of Alb. Mechanistically, we found that a HG environment induced increased mRNA transcription and protein expression of α-SMA, decreased mRNA transcription and protein expression of E-cadherin and PTEN, and increased protein expression of P-Akt, as well as an increasing trend in the P-Akt/Akt protein expression ratio. However, the application of CRT reversed all the aforementioned changes. Additionally, CRT possessed the ability to reduce the protein expression of PI3K and P-Akt, displaying similar efficacy to the known PI3K inhibitor LY29400. LY29400 also reversed the HG-induced alterations in the protein expression of α-SMA, E-cadherin, and PTEN. When CRT and LY29400 were used in combination, they still exhibited the effects of reducing the protein expression of PI3K and P-Akt, as well as the P-Akt/Akt expression ratio. However, the combination further increased the mRNA transcription of PTEN under HG conditions, while the protein expression of P-Akt was elevated compared to the use of CRT alone.

The application of CRT to other kidney diseases has been extensively substantiated. Mao verified through *in vitro* experiments that CRT could significantly improve renal function, effectively alleviate kidney damage, and markedly reduce proteinuria levels in mice with membranous nephropathy. This finding provides strong support for the potential use of CRT as a therapeutic agent for membranous nephropathy (Mao et al., 2023). Furthermore, scholars conducted a retrospective study comparing the effects of using ACEI/ARB alone versus combination therapy with CRT. The results indicated that CRT exhibited significant efficacy in treating patients with idiopathic membranous nephropathy and concomitant proteinuria (Xu et al., 2024). Additionally, CRT demonstrated potential in treating rheumatoid arthritis by regulating the PI3K/HIF1α/NOS2 signaling pathway (Wang K. et al., 2022). Several studies have highlighted the remarkable efficacy of CRT in treating less common diseases such as childhood Henoch-Schonlein purpura nephritis (HSPN) and mesangioproliferative glomerulonephritis (Zhou et al., 2004; Hongbing and Wei, 2005). Moreover, some small-sample clinical studies have also showcased the promising potential of CRT in the treatment of DKD, which is consistent with our research findings and further reinforces the reliability of our results (Wang L, 2015; Wang X, 2016; Zhou J, 2016).

DKD, primarily characterized by diabetic nephropathy (DN), typically manifests as impaired renal function or increased urinary albumin excretion (Anders et al., 2018). Additionally, clinical manifestations of DKD may include renal artery atherosclerosis, renal arteriolar sclerosis, and acute kidney injury episodes associated with heart disease (Umanath and Lewis, 2018). Current standard treatment approaches include strict blood glucose control, nutritional support, physical exercise, and health education (Ismail-Beigi et al., 2010; Bullock et al., 2017). Pharmacological treatment usually involves ACEI or ARBs, with evidence suggesting their ability to reduce DKD progression in patients with macroalbuminuria (Brenner et al., 2001). Despite these measures, the expected outcomes for DKD patients remain suboptimal, with many facing life-threatening complications before their condition progresses to ESRD (Adler et al., 2003).

Several studies have explored the potential mechanisms of CRT in the treatment of DKD. An et al. analyzed common targets and key pathways and found that CRT may improve DKD through anti-nephritis, anti-renal fibrosis, antioxidant, and podocyte protection effects (An et al., 2022). Other studies have discovered that CRT inhibits CD36 expression both *in vivo* and *in vitro* and activates the AMPK pathway, collectively promoting autophagy and inhibiting apoptosis in DKD (Li et al., 2023). The PI3K/Akt signaling pathway plays a crucial role in cell proliferation, differentiation, and metabolism. CRT may become a candidate drug for the treatment of DKD by reversing the imbalance in the immune-inflammatory system mediated by the PI3K/AKT/NF-κB/IL-1β/TNF-α signaling pathway (Ma et al., 2022).

EMT is one of the crucial mechanisms leading to increased urinary albumin excretion. In the early stages of EMT, epithelial cells lose their adhesive properties due to the downregulation of E-cadherin expression, resulting in the disruption of cell-cell junctions. Concurrently, the increased expression of α-SMA, which is associated with alterations in cell morphology and migratory capacity, enables cells to acquire enhanced motility and invasiveness (Yang and Liu, 2001). During the EMT process, renal tubular epithelial cells transform into cells with mesenchymal characteristics, leading to the loss of cellular barrier function and consequently augmenting albumin leakage (Maggiorani et al., 2015).

EMT is a highly complex biological process, initially proposed by Elizabeth Hay and colleagues in 1982 (Greenburg and Hay, 1982). It is closely associated with various aspects, including tumor progression and metastasis, tissue fibrosis, and wound healing (Jonckheere et al., 2022). HK-2 cells are a crucial cell type in the renal tubulointerstitium. Under diabetic conditions, these cells lose their inherent cellular characteristics, exhibit enhanced motility and migratory capabilities, and acquire mesenchymal cell features. This phenomenon further leads to the abnormal accumulation of ECM in the renal interstitium, culminating in renal tubulointerstitial fibrosis and ultimately resulting in renal dysfunction and the development of end-stage renal disease. This process is gradual, sequential, and irreversible. In our study, we observed that the morphology of normal HK-2 cells predominantly resembled cobblestones. However, following a 48-h intervention with HG, the cells adopted a more spindle-shaped appearance with increased protrusions and disorganized cellular arrangement, which is consistent with previous findings (Griggs et al., 2017). Furthermore, in this study, CRT reversed the abnormal expression of EMT biomarkers, specifically α-SMA and E-cadherin, in HG-induced HK-2 cells, suggesting that CRT may inhibit the occurrence of EMT.

EMT plays a pivotal role throughout the development and progression of DKD and is closely associated with renal tubulointerstitial fibrosis. Consequently, inhibiting tubular EMT may be a key to preventing renal interstitial fibrosis. To date, numerous studies have demonstrated that the PI3K/AKT signaling pathway plays a crucial role in EMT during DKD. The hyperglycemic internal environment in diabetic patients leads to the aberrant activation of this signaling pathway, resulting in substantial ECM deposition in renal tissues and inducing EMT in HK-2 cells, thereby accelerating the process of renal fibrosis (Xie et al., 2015). PTEN is a well-recognized negative regulator of the PI3K/Akt pathway. During signal transduction, PTEN degrades phosphatidylinositol (3,4,5)-trisphosphate to phosphatidylinositol bisphosphate, thereby counteracting the actions of PI3K and exerting a range of physiological functions (Maehama and Dixon, 1998; Sulis and Parsons, 2003). Research has revealed that PTEN plays a significant role in the fibrotic processes of various organs. Scholars have discovered through extensive experiments that increasing PTEN expression can alleviate liver fibrosis in rats (Lok et al., 2020). Kahori Miyoshi et al. suggested that PTEN can attenuate acute lung injury and delay pulmonary fibrosis by inhibiting the EMT process in alveolar epithelial cells (Miyoshi et al., 2013). Kattla et al. demonstrated that PTEN exerts antifibrotic and renoprotective effects by negatively regulating the PI3K/Akt pathway (Kattla et al., 2008). However, it remains unclear whether the PTEN/PI3K/AKT signaling pathway is involved in the anti-EMT effects of CRT in HK-2 cells. Our research findings indicate that under HG intervention, PTEN expression decreases and PI3K/Akt pathway activity increases. Upon application of the PI3K inhibitor LY29400, PTEN expression was observed to increase, and PI3K/Akt pathway activity was inhibited. LY29400 can influence HG-induced EMT in HK-2 cells by suppressing Akt activation, which is consistent with previous research results by Liang, Zhang, and others (Liang et al., 2015; Zhang et al., 2015). Following intervention with CRT, PTEN expression was similarly promoted, PI3K/AKT pathway inactivation was enhanced, and the transformation of HK-2 cells into mesenchymal cells was prevented, potentially exerting antifibrotic and renoprotective effects. Therefore, we discovered that the PTEN/PI3K/AKT pathway might be the potential mechanism underlying CRT’s ability to alleviate HG-induced EMT in HK-2 cells.

### 4.1 Limitations

While our study provides valuable insights into the efficacy and mechanisms of CRT in the treatment of DKD, several limitations should be acknowledged. First, the sample size of our clinical trial was relatively small, which may limit the generalizability of our findings. Future studies with larger patient cohorts are needed to validate our results. Second, the follow-up period of our clinical trial was relatively short (3 months), which may not be sufficient to assess the long-term effects of CRT on DKD progression and patient outcomes. Long-term follow-up studies are warranted to evaluate the sustained efficacy and safety of CRT in DKD patients. Third, our *in vitro* experiments were conducted using a single cell line (HK-2), which may not fully recapitulate the complex pathophysiology of DKD. Future studies should employ additional cell lines and animal models to further investigate the mechanisms of CRT in the context of DKD. Lastly, while we identified the PTEN/PI3K/AKT pathway as a potential mechanism underlying the anti-EMT effects of CRT, other signaling pathways and molecular targets may also be involved. Comprehensive mechanistic studies are needed to fully elucidate the therapeutic mechanisms of CRT in DKD.

## 5 Conclusion

In conclusion, our study demonstrates that CRT is an effective treatment for DKD, as evidenced by its ability to significantly improve proteinuria and increase the effective treatment rate in DKD patients. Furthermore, our *in vitro* experiments reveal that CRT can ameliorate HG-induced EMT in HK-2 by upregulating PTEN expression and inhibiting the PI3K/AKT signaling pathway. These findings provide new insights into the therapeutic potential and underlying mechanisms of CRT in the treatment of DKD. The ability of CRT to target EMT, a crucial process in the development and progression of renal fibrosis, highlights its promise as a novel therapeutic agent for DKD. Future studies should focus on further elucidating the molecular mechanisms of CRT and exploring its long-term efficacy and safety in larger clinical trials.

## Data Availability

The original contributions presented in the study are included in the article/Supplementary material, further inquiries can be directed to the corresponding author.
